# Effects of rituximab dose on hepatitis B reactivation in patients with resolved infection undergoing immunologic incompatible kidney transplantation

**DOI:** 10.1038/s41598-018-34111-5

**Published:** 2018-10-23

**Authors:** Juhan Lee, Jun Yong Park, Deok Gie Kim, Jee Youn Lee, Beom Seok Kim, Myoung Soo Kim, Soon Il Kim, Yu Seun Kim, Kyu Ha Huh

**Affiliations:** 1 Department of Transplantation Surgery, Severance Hospital, Yonsei University Health System, Seoul, Republic of Korea; 2 Department of Gastroenterology, Severance Hospital, Yonsei University Health System, Seoul, Republic of Korea; 3 Department of Nephrology, Severance Hospital, Yonsei University Health System, Seoul, Republic of Korea; 4 Department of Surgery, Yonsei University College of Medicine, Seoul, Republic of Korea

## Abstract

Sensitized patients received desensitization therapy with rituximab for kidney transplantation. However, the impact of rituximab dose on hepatitis B virus (HBV) reactivation is unknown. Patients who underwent living donor kidney transplantation between 2008 and 2016 were grouped according to rituximab dose (control vs. standard-dose rituximab [375 mg/m^2^] vs. reduced-dose rituximab [200 mg/body]) for comparison of HBV reactivation. A total of 336 hepatitis B surface antigen (HBsAg)-negative/antibody to hepatitis B core antigen (anti-HBc)-positive patients underwent kidney transplantation, of whom 91 (27.1%) received rituximab for desensitization (57 standard-dose and 34 reduced-dose rituximab). During the study period, eight patients experienced HBV reactivation (three in the control group, five in the standard-dose group). In the standard-dose group, four patients experienced hepatitis flare, and one patient died due to hepatic failure. No HBV reactivation occurred in the reduced-dose group. Standard-dose rituximab significantly decreased hepatitis B surface antigen antibody titer (anti-HBs; −99.8 IU/L) at 12 months, compared with reduced-dose rituximab (−20.1 IU/L) and control (−39.1 IU/L, *P* = 0.017). Standard-dose rituximab (HR, 10.60; 95% CI, 2.52–44.60; *P* = 0.001) and anti-HBs < 100 IU/L at transplantation (HR, 9.06; 95% CI, 1.11–74.30; *P* = 0.04) were independent risk factors for HBV reactivation. Standard-dose rituximab significantly increased HBV reactivation risk for HBsAg-negative/anti-HBc-positive kidney transplant patients.

## Introduction

Hepatitis B virus (HBV) infection is the common chronic viral infection in the world^1^. Although the prevalence is decreasing as a result of vaccination, roughly 30% of the world’s population shows serological evidence of resolved HBV infection (hepatitis B surface antigen [HBsAg]-negative/antibody to hepatitis B core antigen [anti-HBc]-positive). Although safe and effective antiviral drugs are available to prevent HBV reactivation, fatal HBV reactivations have occurred in resolved HBV patients, particularly after receiving rituximab therapy^2–4^.

In kidney transplant, desensitization with rituximab allows patients who are sensitized to human leukocyte antigen or blood antigen to overcome the immunologic barrier^5,6^. When introduced in the transplant setting, the rituximab standard dose (375 mg/m^2^) was based on the treatment of lymphoma^7^. The effect of reduced rituximab doses (10–300 mg/m^2^) has been tested sufficiently on splenic and peripheral blood B cells^8^. In addition, a Japanese group reported favorable long-term outcomes of ABO-incompatible (ABOi) kidney transplantation with a reduced dose of rituximab (a fixed dose of 200 mg)^9^. Thereafter, many transplant centers began to use reduced doses of rituximab to improve safety and cost-effectiveness.

Dose intensity of rituximab has previously been found to be an important risk factor for HBV reactivation in patients with hematologic malignancy^10^. In the transplant setting, however, rituximab is generally administered as a single course with different doses^11^. Although more than 20% of kidney transplant recipients receive rituximab for desensitization, only limited data regarding rituximab dose and subsequent HBV reactivation are available for this population^12,13^. Consequently, we directly compared the impact of rituximab dose on HBV reactivation after kidney transplantation.

## Results

### Patients

**Figure 1:**
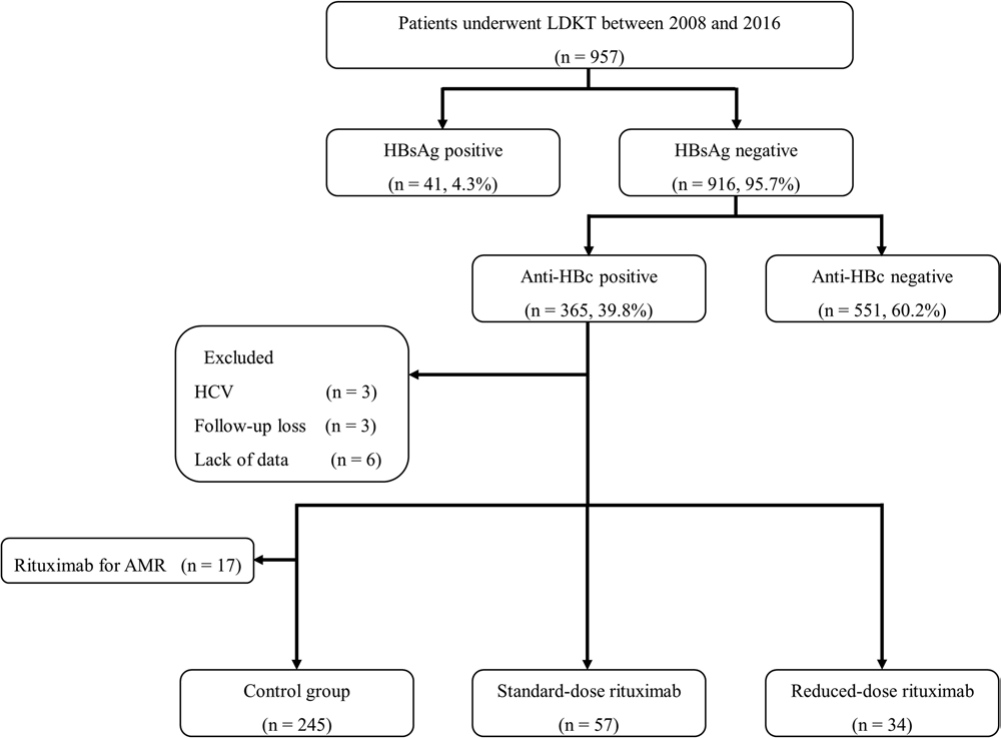
Study design.

A total of 957 patients underwent living donor kidney transplantation between 2008 and 2016. Of them, 365 (38.1%) were HBsAg-negative/anti-HBc-positive at the time of transplantation. During the study period, 262 patients received kidney transplantation without rituximab desensitization and 91 received rituximab desensitization. Of the 262 patients who did not receive rituximab, 17 were excluded due to the use of rituximab post-transplant (Fig. 1).

### Baseline characteristics

Baseline characteristics of patients are presented in Table 1. There were no significant differences in age or dialysis among the groups; however, the proportion of female patients and re-transplant cases, and the mean number of human leukocyte antigen mismatches were significantly higher in the standard- and reduced-dose rituximab groups than in the control group. However, there were no significant differences in sex, re-transplant, and mean number of HLA mismatches between standard- and reduced-dose rituximab groups. Sero-positivity of anti-HBs (≥10 IU/L) and donor anti-HBc were comparable among the groups; however, there was a significant difference in the proportion of anti-HBs ≥100 IU/L, which was significantly lower in the reduced-dose rituximab group. The use of ATG for induction was significantly more common for patients in the reduced- and standard-dose rituximab group than for patients in the control group, but the use of ATG for anti-rejection therapy was not significantly different among the groups. The use of cyclosporin for maintenance immunosuppression was significantly more common in the control group than in the other groups. In the standard-dose rituximab group (375 mg/m^2^), mean dose of rituximab was 600 mg, which was three times that of the reduced-dose rituximab group (200 mg). Median follow-up times for the control, standard-dose, and reduced-dose rituximab groups were 74, 65, and 31.5 months, respectively. The date of last patient follow-up was December 15, 2017.

**Table 1:** Baseline characteristics.

Variables	Control (n = 245)	Standard dose rituximab (n = 57)	Reduced dose rituximab (n = 34)	*P*-value
Age (years)	50.0 ± 10.4	49.6 ± 7.9	54.0 ± 8.3	0.072
Female, n (%)	75 (30.6%)	24 (42.1%)	18 (52.9%)	0.017
Duration of dialysis (months)	15.4 ± 30.4	22.0 ± 42.9	14.6 ± 22.3	0.367
HLA mismatch number	2.9 ± 1.5	3.7 ± 1.6	3.5 ± 1.3	<0.001
Retransplantation, n (%)	10 (4.1%)	10 (17.5%)	7 (20.6%)	<0.001
Anti-HBs positive (≥10 IU/L), n (%)	209 (85.3%)	50 (87.7%)	30 (88.2%)	0.827
Anti-HBs ≥ 100 IU/L, n (%)	113 (46.1%)	33 (57.9%)	10 (29.4%)	0.03
Donor anti-HBc positive, n (%)	84 (34.3%)	20 (35.1%)	8 (23.5%)	0.438
Induction agents				<0.001
Basiliximab, n (%)	243 (99.2%)	49 (86.0%)	21 (61.8%)	
ATG, n (%)	2 (0.8%)	8 (14.0%)	13 (38.2%)	
ATG for anti-rejection	9 (3.7%)	8 (14.0%)	1 (2.9%)	0.226
Maintenance CNI				0.001
Tacrolimus, n (%)	192 (78.4%)	55 (96.5%)	32 (94.1%)	
Cyclosporin, n (%)	53 (21.6%)	2 (3.5%)	2 (5.9%)	
Trough level of tacrolimus (at 1 year)	5.6 ± 3.0	5.2 ± 3.2	5.8 ± 1.9	0.563
Trough level of tacrolimus (at 2 year)	5.0 ± 1.9	5.2 ± 3.7	5.8 ± 3.0	0.281
Number of plasmapheresis	—	3.96 ± 3.23	4.24 ± 4.26	0.248
Median follow-up (months)	74 [44.5, 99]	65 [48, 78.5]	31.5 [17.8, 40]	<0.001

### HBV reactivation

During the study period, eight patients experienced HBV reactivation. Of these, five cases occurred in the standard-dose rituximab group (5/57, 8.8%), and three cases occurred in the control group (3/245, 1.2%). However, no HBV reactivation occurred in the reduced-dose rituximab group. In the standard-dose rituximab group, the median time from rituximab desensitization to HBV reactivation was 11 months (range, 5–22 months). For the three patients with HBV reactivation, the median time from kidney transplantation to HBV reactivation was 48 months (range, 24–57 months). With respect to the pre-transplant anti-HBs titers in patients with HBV reactivation, seven patients had low titers of anti-HBs (<100 IU/L), whereas one patient had a high titer of anti-HBs (≥100 IU/L).

### Clinical outcomes

The clinical features of the eight patients with HBV reactivation are listed in Table 2. Five patients (A–E) received a single dose of rituximab 2–7 days before kidney transplantation. At the time of HBV reactivation, all patients received maintenance therapy with a triple immunosuppressant regimen consisting of tacrolimus, prednisone, and mycophenolate mofetil (MMF). Two patients (C and E) were treated for acute rejection 21 and 8 months prior to HBV reactivation, respectively. Among the five patients described, four experienced hepatitis flare (serum ALT >100 IU/L) at the time of HBV reactivation.

**Table 2:** Clinical outcomes of HBV reactivation.

Patient	Sex	Age (years)	Baseline anti-HBs (IU/L)	Rituximab (cause)	Anti-rejection therapy prior to reactivation	Time to reactivation after KT (months)	HBV DNA (IU/mL) at diagnosis	Peak ALT (U/L)	Outcomes
A	M	48	265.65	375 mg/m^2^ (ABOi)		11	>1.7 × 10^8^	641	Alive with functioning graft
B	M	59	13.56	375 mg/m^2^ (ABOi)		5	>1.7 × 10^8^	340	Death due to liver failure
C	M	48	52.08	375 mg/m^2^ (XM+)	ACR	22	1.23 × 10^7^	39	Alive with functioning graft
D	M	61	Negative (3.99)	375 mg/m^2^ (ABOi)		5	>1.7 × 10^8^	524	Death due to unknown causes
E	M	51	Negative (0.63)	375 mg/m^2^ (XM+)	AMR	12	4.94 × 10^7^	237	Alive with functioning graft
F	M	61	Negative (1.22)	No		24	>1.7 × 10^8^	213	Alive with functioning graft
G	M	60	10.91	No		48	5.22 × 10^7^	52	Alive with functioning graft
H	M	49	73.59	No		57	5.32 × 10^7^	50	Alive with functioning graft

Three patients (F–H) in the control group experienced HBV reactivation. All three patients received maintenance therapy with tacrolimus and prednisone. Two of these patients also received MMF, and the other patient received mTOR inhibitor therapy as well. None of the patients received anti-rejection therapy prior to HBV reactivation. Among the three patients, one experienced hepatitis flare at the time of HBV reactivation.

All patients initiated entecavir upon detection of reactivation. One patient in the standard-dose rituximab group (patient B) died of hepatic failure despite active antiviral treatment. Another patient in the standard-dose rituximab group (patient D) died due to unknown causes 20 months after HBV reactivation.

### Risk factors for HBV reactivation

Factors associated with HBV reactivation were analyzed using a Cox regression model. As shown in Table 3, anti-HBs < 100 IU/L at the time of transplantation and standard-dose rituximab for desensitization were significant independent risk factors for HBV reactivation. The causes of rituximab (ABO incompatible, positive crossmatch, or high PRA) were not associated with HBV reactivation.

**Table 3:** Risk factors for HBV reactivation.

	Univariate		Multivariate	
	HR (95% CI)	P-value	HR (95% CI)	P-value
Rejection	2.731 (0.653, 11.429)	0.169		
Use of ATG	1.509 (0.183, 12.429)	0.702		
Standard dose rituximab	8.256 (1.973, 34.551)	0.004	10.598 (2.519, 44.599)	0.001
anti-HBs ≥ 100 IU/L	0.149 (0.018, 1.214)	0.075	0.11 (0.013, 0.905)	0.04
Donor anti-HBc (+)	0.652 (0.132, 3.230)	0.600		
Causes of rituximab	0.885 (0.148, 5.297)	0.893		
Number of plasmapheresis	1.343 (0.862, 1.913)	0.314		
Tacrolimus trough level	0.899 (0.652, 1.240)	0.516		
MMF dose	1.001 (0.999,1.002)	0.280		

During the study period, 15 patients of the standard-dose rituximab group (26.3%) and 8 patients of the reduced-dose rituximab group (23.5%) received plasmapheresis ± intravenous immunoglobulin for treatment of antibody-mediated rejection (AMR, *P* = 0.767). AMR treatment was not associated with HBV reactivation.

**Figure 2:**
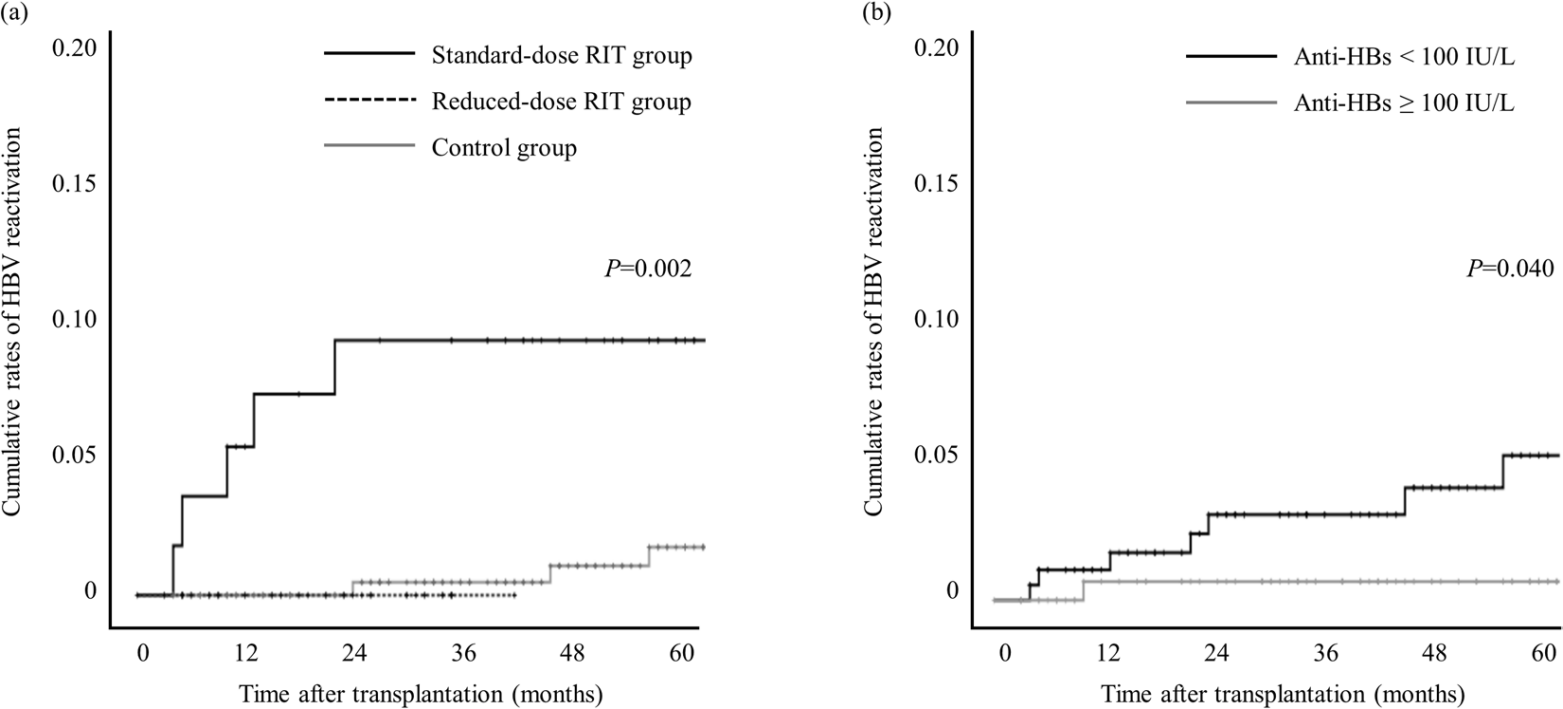
Cumulative rates of HBV reactivation. (**a**) HBV reactivation according to rituximab dose. (**b**) HBV reactivation according to anti-HBs titers at the time of transplantation (anti-HBs < 100 IU/L vs. anti-HBs ≥ 100 IU/L).

The cumulative rates of HBV reactivation, depending on rituximab desensitization and anti-HBs status, are shown in Fig. 2.

### Anti-HBs levels during follow-up

**Figure 3:**
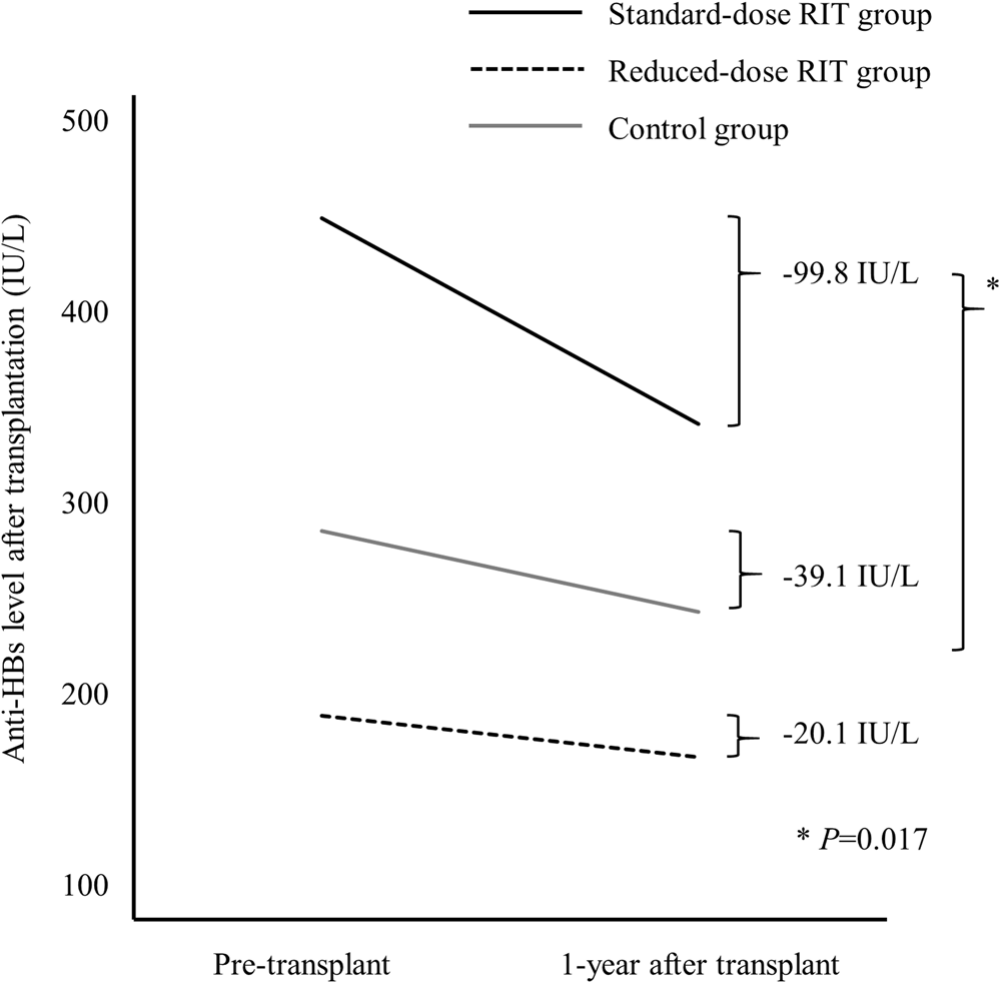
Changes of anti-HBs titer according to rituximab dose.

The changes in anti-HBs levels from pre-transplant measurement and 1-year after transplantation are shown in Fig. 3. Overall, anti-HBs titers decreased during the first year after transplantation regardless of treatment group. At 1-year after kidney transplantation, the mean changes in anti-HBs between the standard-dose rituximab, reduced-dose rituximab, and control groups were −99.8, −20.1, and −39.1 IU/L, respectively (*P* = 0.017). *Post-hoc* analysis indicated that standard-dose rituximab significantly reduced the anti-HBs titer.

The mean numbers of plasmapheresis sessions were similar between the standard- and reduced-dose rituximab groups. One-third of patients received five or more sessions of plasmapheresis (20/57 of patients in the standard-dose rituximab group and 8/34 patients in the reduced-dose rituximab group). Evaluation of the impact of plasmapheresis (five or more sessions) on anti-HBs titers revealed no difference between the groups.

## Discussion

Rituximab, a human-mouse chimeric monoclonal antibody that targets B cells, has significantly improved clinical outcomes in B-cell lymphoma patients and has been increasingly used in organ transplantation and autoimmune diseases^5,6,14,15^. Despite its potent cytolytic effect, rituximab is generally well tolerated with minimal toxicity; however, increasing evidence indicates that rituximab is associated with HBV reactivation^16^. Therefore, the US Food and Drug Administration (FDA) issued a new boxed warning that rituximab increases the risk of HBV reactivation^17^.

Fatal HBV reactivation can occur not only in patients with hematologic malignancy but also in patients with kidney transplantation, particularly after receiving rituximab therapy^2–4,12^. In this study, the risk of HBV reactivation and its severity were found to be associated with the dose of rituximab. Our findings indicate that a standard dose of rituximab (375 mg/m^2^) increases the risk of HBV reactivation in HBsAg-negative/anti-HBc-positive kidney transplant patients. In contrast, a reduced dose of rituximab (200 mg/body) did not increase the risk of HBV reactivation.

The mechanism of rituximab-associated HBV reactivation is not fully understood^11^. Although control of HBV infection is mediated mainly by HBV-specific cytotoxic T cells, B cells are still required for antigen-presentation. B-cell depletion by rituximab may disrupt CD8 + cytotoxic T cell killing of HBV-infected hepatocytes^18^. In addition, rituximab administration changes T lymphocyte activity by increasing Th1/Th2 and Tc1/Tc2 ratios and up-regulating the Fas ligand on Th1 and Th2 cells^19^.

Considering the net state of immunosuppression, the dose of rituximab may significantly affect HBV reactivation^20^. Prior studies demonstrated that patients with advanced malignancy had a higher risk for HBV reactivation, possibly because these patients received more cycles of chemotherapy. In contrast, patients with limited-stage lymphoma who received fewer cycles of rituximab experienced a reduction in HBV reactivation^10^. In line with previous studies, our data indicate that a standard-dose rituximab significantly increase the risk of HBV reactivation. In addition, standard dose of rituximab may be related to the severity of liver damage^21^. Among the five patients who received a standard dose of rituximab and experienced HBV reactivation, four experienced hepatitis flare, including one patient who died from hepatic failure. In contrast, of the three patients with HBV reactivation in the control group, only one developed hepatitis flare and none of them died from hepatic failure.

In contrast to patients receiving standard-dose rituximab, none of the patients receiving reduced-dose rituximab developed HBV reactivation. Recently, Masutani *et al*. also founded no significant association between HBV reactivation and a reduced dose of rituximab in kidney transplant patients^13^. However, such previous studies, including ours, compared a rituximab group with a control group, limiting the interpretation of the effects of rituximab dose on the outcomes^12,13^. The current study design allows us to assess the effect of rituximab dose on HBV reactivation more clearly.

The protective role of anti-HBs against HBV reactivation in patients receiving rituximab therapy is controversial^22^. The FDA reported that HBV reactivation also occurred in patients with anti-HBs^20^, while other studies showed a protective effect of anti-HBs in patients receiving rituximab-based chemotherapy^3,4,23^. In this study, we found that high titers of anti-HBs (≥100 IU/L) at the time of transplantation significantly decreased the risk of HBV reactivation (HR, 0.11). In contrast, one patient with a high titer of anti-HBs experienced HBV reactivation 10 months after receiving a standard dose of rituximab. Accordingly, anti-HBs at the time of rituximab may provide a protective effect against HBV reactivation, but this association is not absolute^23,24^.

Another key finding of this study is that rituximab decreases anti-HBs titer in a dose-dependent manner. Although there were differences in baseline anti-HBs titers among groups, our results support the idea that a standard dose of rituximab increases the risk of HBV reactivation. Therefore, pre-transplant screening for anti-HBs should be included in conjunction with HBsAg and anti-HBc^25,26^. In patients with low levels of anti-HBs, pre-transplant HBV vaccination prior to standard-dose rituximab treatment should be considered. Future studies should examine the potential role for booster vaccinations and antiviral prophylaxis according to anti-HBs titers^23^.

Unfortunately, a consensus has not been reached for prophylaxis or monitoring in resolved HBV patients^25,26^. In addition, evaluation of cost-effectiveness is much more complex in an organ transplant setting that require life-long immunosuppressive therapy. Although the prevalence of HBV differs across the country, HBV reactivation does occur, and the risk is likely clinically significant^27^. In this study, the median time from standard-dose rituximab desensitization to reactivation was 11 months. Of note, one case of reactivation occurred 22 months after rituximab desensitization. On the other hands, the risk of HBV reactivation in the reduced-dose group was similar to that in the control group. Our findings suggest that prophylactic antiviral therapy or regular DNA monitoring (every 3 months) for 24 months after renal transplantation is important in resolved HBV patients receiving a standard dose of rituximab^28^.

There are several limitations to this study. First, the number of patients with HBV reactivation may have been underestimated. The intensity of monitoring can influence the resulting incidence of HBV reactivation^22^. Although ALT and biochemical tests were performed every month for the first year post-transplant, HBV markers were tested only annually or in patients with elevated ALT. Thus, some asymptomatic HBV reactivation episodes may have been overlooked. Second, as the desensitization protocol was modified recently, the follow-up duration varies among the groups. Although some late HBV reactivation cases have been reported (up to 33 months after rituximab treatment), most HBV reactivation occurs within one year after rituximab treatment. Thus, we think follow-up duration (31.5 months) is sufficient time to see HBV reactivation after reduced-dose rituximab desensitization^29^. Third, we noted an imbalance of immunological risk factors among groups, as rituximab dose is determined based on immunological risk. However, HBV reactivation is affected by the “net state of immunosuppression”, not by an immunological risk factor itself. Except for desensitization, we maintained similar maintenance immunosuppressive regimens and trough levels throughout the study period.

In conclusion, the risk of HBV reactivation in HBsAg-negative/HBcAb-positive patients is significantly related to the dose of rituximab. Standard-dose rituximab (375 mg/m^2^) for desensitization significantly increases the risk of HBV reactivation and hepatitis flare and decreases the anti-HBs titer, compared with reduced-dose rituximab (200 mg/body) and no rituximab. Close monitoring of HBV DNA and anti-HBs, as well as, the prophylactic or preemptive use of antiviral agents should be considered for these patients.

## Materials and Methods

### Subjects

A total of 957 adult patients who underwent living donor kidney transplantation between 2008 and 2016 at Severance Hospital in Seoul, Korea were screened. Patients who were both HBsAg-negative and anti-HBc-positive were selected. We excluded patients with hepatitis C virus (HCV), follow-up loss, and lack of data. Patients were categorized into standard-dose rituximab (375 mg/m^2^), reduced-dose rituximab (200 mg/body), and control (no rituximab) groups. In the control group, we excluded patients who were receiving rituximab for AMR treatment (Fig. 1). All donor surgeries were performed at the same hospital with the consent of the donor and approval from the Korean Network Organ Sharing. No allografts (organs and tissue) obtained from prisoners were used.

### Ethical approval

Written informed consent was obtained from kidney transplant recipients. The study procedures were conducted in accordance with the Declaration of Helsinki and approved by the Institutional Review Board of Severance Hospital (4-2014-1105).

### Definitions

We defined resolved HBV infection as HBsAg-negative/anti-HBc-positive patients without HBV DNA at the time of transplantation^4,30^. HBV reactivation was defined as the reemergence of HBsAg (HBsAg seroreversion) or detectable HBV DNA in serum^31^.

Hepatitis flare was defined as ≥3-fold increase in serum alanine aminotransferase (ALT) levels that exceeded 100 IU/L. HBV-related hepatitis flare was defined as hepatitis flare with HBV reactivation, in the absence of laboratory features of acute infection with hepatitis A virus, HCV, or cytomegalovirus^2^.

### Monitoring and management of HBV reactivation

All patients were screened for HBV (HBsAg, antibody to hepatitis B surface antigen [anti-HBs], anti-HBc, and HBV DNA) and HCV before transplantation. The follow-up protocol included the following: (1) routine biochemical tests (including ALT) every month for the first year post-transplant and then every 3 months thereafter, (2) assessment of yearly HBV markers (HBsAg and anti-HBs), (3) assessment of additional HBsAg for patients with hepatitis flare (serum ALT > 100 IU/L), and (4) detection of HBV DNA in cases of HBsAg seroreversion and/or ALT elevation.

Serum HBsAg, anti-HBc, and anti-HBs were evaluated using commercially available enzyme immunoassays (Abbott Diagnostics, Abbott Park, IL, USA). Titers of serum anti-HBs < 10 IU/L were considered negative. Serum HBV DNA was measured using real-time polymerase chain reaction assay on a Cobas TaqMan 48 Analyzer (Roche Molecular Systems, Branchburg, NJ, USA), with 20 IU/mL as the lower limit of detection.

No patients received prophylactic antiviral agents. Entecavir was initiated for patients who experienced HBV reactivation.

### Immunosuppressive regimen

**Figure 4:**
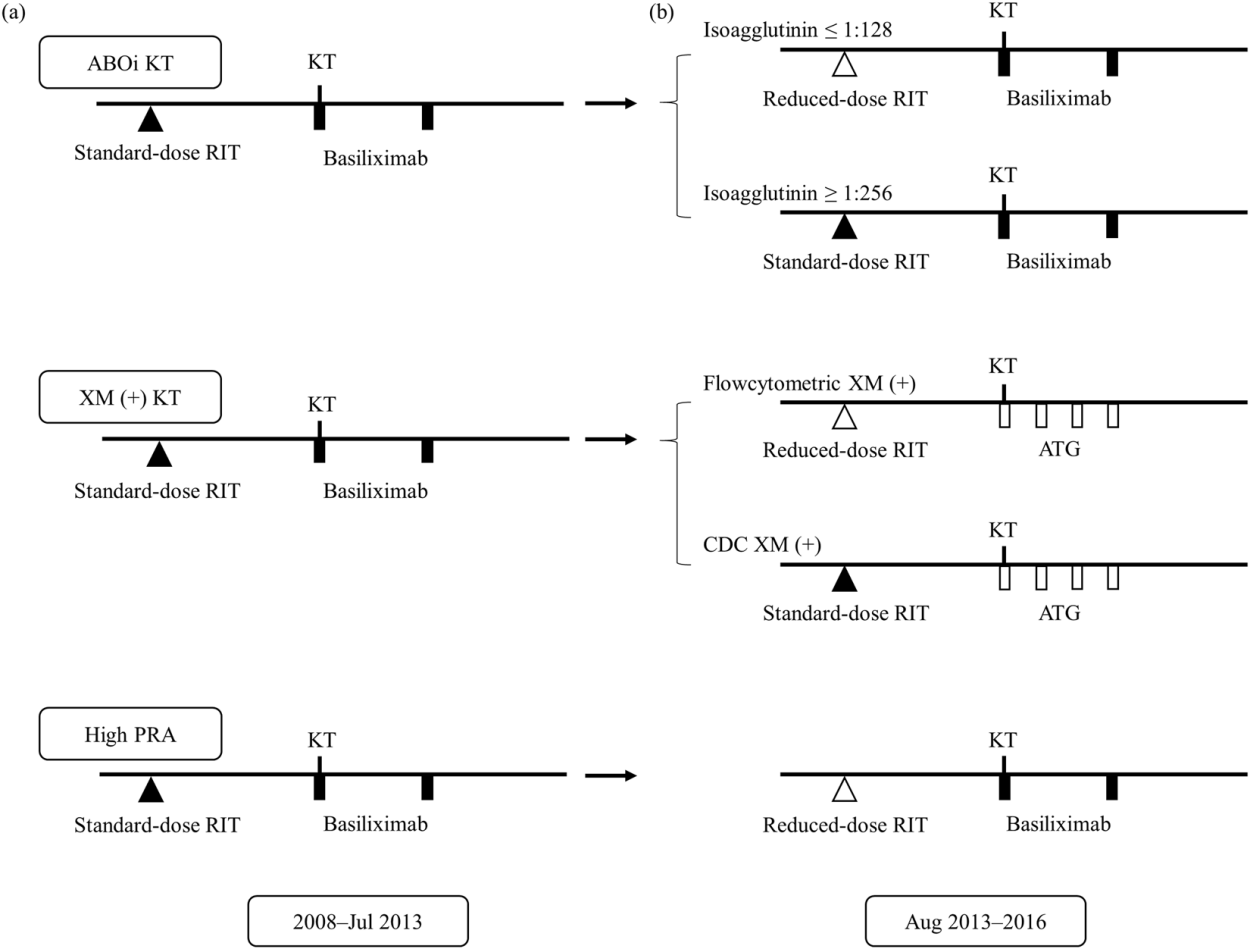
Desensitization protocols for kidney transplantation. (**a**) Desensitization with a standard dose of rituximab (2008–July 2013). (**b**) Modified desensitization with a reduced dose of rituximab (August 2013–2016).

As previously described, rituximab was administered within 7 days before kidney transplantation in cases of ABO-incompatible, positive crossmatch, or high panel reactive antibodies (PRA; having a PRA > 50%)^32^. Since August 2013, our clinic has used a reduced dose of rituximab (200 mg/body) based on the patient’s immunological risk (Fig. 4). Plasmapheresis was performed until the target antibody titer (IgG titer ≤ 1:16 in ABOi KT, conversion of a positive crossmatch to negative) was achieved.

Most patients received induction immunosuppression with basiliximab (20 mg on day 0 and 4). Since 2013, we have used anti-thymocyte globulin (ATG) for induction in positive crossmatch patients (1.5 mg/kg per day for 4 days). The maintenance immunosuppressive regimen mostly consisted of calcineurin inhibitor (cyclosporin or tacrolimus) and prednisolone with or without MMF. MMF was administered 1 week before transplantation in cases of ABOi KT or positive crossmatch KT. We administered calcineurin inhibitor 1 day before or on the day of transplantation in all patients, regardless of immunologic risk. The doses of maintenance immunosuppression were administered per institutional protocols as previously described^29^.

Acute cellular rejection (ACR) was treated using methylprednisolone pulse therapy (500 mg/day, three to four times). Patients with steroid-resistant ACR patients received ATG. AMR was treated with a combination of plasmapheresis and intravenous immunoglobulin with or without rituximab.

### Statistical analysis

Data were expressed as frequency (percentage), mean and standard deviation, or median and interquartile range, depending on data type. Chi-square or Fisher’s exact tests were used as appropriate to compare categorical variables. Continuous variables were compared using one-way analysis of variance. When the data revealed a statistically significant difference, *post hoc* comparisons were performed by applying Bonferroni’s correction for multiple comparisons. Cumulative rates of HBV reactivation were analyzed using the Kaplan-Meier curves and the log-rank test. Univariate and multivariate analyses were performed using Cox proportional hazard regression models to determine risk factors for HBV reactivation. Statistical analyses were performed using SPSS software (version 23.0; SPSS Inc., Chicago, IL, USA), and *P* < 0.05 was considered statistically significant.

## Data Availability

The datasets generated during and/or analyzed during the current study are available from the corresponding author on reasonable request.
